# Identifying the vegetation type in Google Earth images using a convolutional neural network: a case study for Japanese bamboo forests

**DOI:** 10.1186/s12898-020-00331-5

**Published:** 2020-11-27

**Authors:** Shuntaro Watanabe, Kazuaki Sumi, Takeshi Ise

**Affiliations:** 1 Field Science Education and Research Center (FSERC), Kyoto University, Kitashirakawaoiwake-cho, Sakyo-ku, Kyoto 606-8502 Japan; 2 Graduate School of Agriculture, Kyoto University, Kitashirakawaoiwake-cho, Sakyo-ku, Kyoto 606-8502 Japan; 3 Graduate School of Science and Engineering, Kagoshima University, 1-21-40 Korimoto, Kagoshima, 890-0065 Japan

**Keywords:** Convolutional neural network, Vegetation mapping, Google earth imagery

## Abstract

**Background:**

Classifying and mapping vegetation are crucial tasks in environmental science and natural resource management. However, these tasks are difficult because conventional methods such as field surveys are highly labor-intensive. Identification of target objects from visual data using computer techniques is one of the most promising techniques to reduce the costs and labor for vegetation mapping. Although deep learning and convolutional neural networks (CNNs) have become a new solution for image recognition and classification recently, in general, detection of ambiguous objects such as vegetation is still difficult. In this study, we investigated the effectiveness of adopting the chopped picture method, a recently described protocol for CNNs, and evaluated the efficiency of CNN for plant community detection from Google Earth images.

**Results:**

We selected bamboo forests as the target and obtained Google Earth images from three regions in Japan. By applying CNN, the best trained model correctly detected over 90% of the targets. Our results showed that the identification accuracy of CNN is higher than that of conventional machine learning methods.

**Conclusions:**

Our results demonstrated that CNN and the chopped picture method are potentially powerful tools for high-accuracy automated detection and mapping of vegetation.

## Background

Classifying and mapping vegetation are essential tasks in environmental science research and natural resource management [1]. Conventional methods (e.g., field surveys, manual interpretation of aerial photographs), however, are not effective for acquiring vegetation data because they are labor-intensive and often economically expensive. Remote sensing technology offers a practical and economical means to acquire information on vegetation cover, especially over large areas [2]. Because of its ability to perform systematic observations at various scales, remote sensing can potentially enable classification and mapping of vegetation at high temporal resolutions.

Detection of discriminating visual features is one of the most important steps in almost all computer vision problems, including in the field of remote sensing. Because conventional methods such as support vector machines [3] require hand-designed, time-consuming feature extraction, substantial efforts have been dedicated toward the development of methods for the automatic extraction of features. Recently, deep learning has become a new solution for image recognition and classification because this new method does not require the manual extraction of features.

Deep learning [4, 5] is a type of machine learning technique that uses algorithms inspired by the structure and function of the human brain, called artificial neural networks. Deep learning involves the learning of features and classifiers simultaneously, and uses training data to categorize image content without a priori specification of image features. Among all deep learning-based networks, the convolutional neural network (CNN) is the most popular for learning visual features in computer vision applications, including remote sensing. Recent research has shown that CNN is effective for diverse applications [4–7]. Given its success, CNN has been used intensively in several distinct tasks in various academic and industrial fields, including plant science. Recent research has shown that the CNN can successfully detect plant diseases and accurately classify plant specimens in an herbarium [8–10].

CNN is a promising technology in the field of remote sensing as well [11, 12]. In recent years, CNNs have started to be used for scene tagging and object detection in remote sensing images. Most previous research employed the supervised learning method and suggested that CNNs can accurately detect or classify the objects in remote sensing images. However, the number of studies that use CNNs for detecting or classifying vegetation in remote sensing images is still limited. Li et al. [13] successfully detected oil palm trees with an accuracy exceeding 96% using CNNs. Recently, Guirado et al. [14] used CNNs to detect a wild shrub (*Ziziphus lotus*) that has a wide range of shapes and sizes, from Google Earth images. They demonstrated that CNNs can successfully detect *Ziziphus lotus* and provide better results than conventional object detection methods. However, the detection targets in the existing works are vegetation distributed in a patch form; therefore, detection or classification of vegetation that has ambiguous and amorphous shapes, such as clonal plants, is still challenging. Semantic segmentation [15] is a possible solution to address this challenge; nevertheless, the application of semantic segmentation for vegetation classification is still limited because this approach requires enormous amounts of labeled data based on pixelwise reference maps [16].

Recently, Ise et al. [17] developed an alternative method (chopped picture method) to conveniently classify ambiguous and amorphous objects. This method dissects the images into numerous small squares and efficiently produces the training images. By using this method, Ise et al. [17] correctly classified three moss species and “non-moss” objects in test images with an accuracy of 95%. However, this method has been applied only to high-resolution images, and its applicability to low-resolution images, such as remote sensing images, has not yet been investigated.

In this study, we investigated the efficiency of adopting a deep learning model and the chopped picture method for computer-based vegetation detection from Google Earth images. We used bamboo forests as the target for vegetation detection. In recent years, bamboo has become invasive in Japan. The bamboo species moso (*Phyllostachys edulis*) and madake (*Phyllostachys reticulata*) are the two main types of exotic bamboo. Since the 1970s, the bamboo industry in Japan has declined as a result of cheaper bamboo imports and heavy labor costs [18]. Consequently, many bamboo plantations were left unmanaged, eventually leading to the invasion of adjacent native vegetation [19–21].

In this study, we specifically addressed the following research questions: (1) how does the resolution of images affect the accuracy of detection; (2) how does the chopping size of training images affect the accuracy of detection; and (3) can a model that was trained for one geographical location work well for a different location?

## Materials and methods

### Target area and image acquisition

**Fig. 1:**
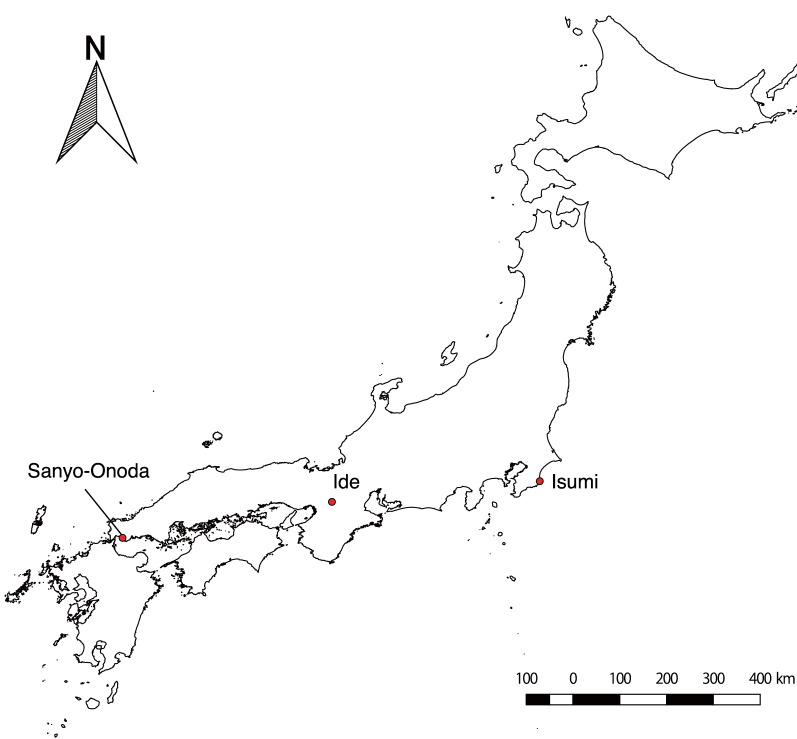
Target regions of this research. This figure was generated using data Global Map Japan version 2.2 (Geospatial Information Authority of Japan)

In this study, we chose three regions (Sanyo-Onoda, Ide, and Isumi) in Japan to perform the analyses (Fig. 1). We used Google Earth as the source of imagery. Google Earth images have various data sources, ranging from medium-resolution Landsat images to high-resolution QuickBird satellite images. Herein, we used high-resolution QuickBird satellite images (spatial resolution: 0.65 m/pixel). From a given sampling location, we obtained the images at zoom levels of 1/500 (spatial resolution: ~ 0.13 m/pixel), 1/1000 (spatial resolution: ~ 0.26 m/pixel), and 1/2500 (spatial resolution: ~ 0.65 m/pixel). Each study site was imaged from Google Earth images during October 2014 (Sanyo-Onoda), May 2017 (Ide), and January 2017 (Isumi).

### Methods and background concepts for the neural networks

**Fig. 2:**
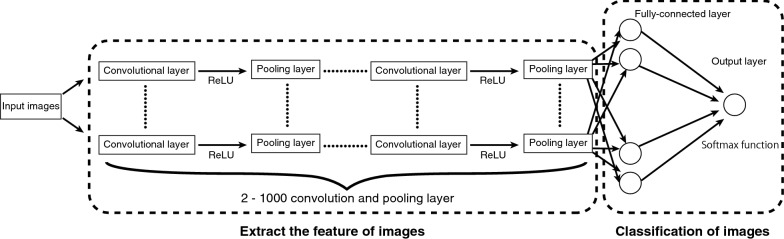
Schematic of the convolutional neural networks

In this study, we employed CNNs (Fig. 2), which are a special type of feedforward neural networks that consist of several convolutional layers and pooling layers.

A feedforward neural network is an artificial neural network wherein connections between the nodes do not form a cycle. These networks, which perform modeling similar to the neuron activity in the brain, are generally presented as systems of interconnected processing units (artificial neurons) that can compute values from inputs, resulting in an output that may be used on further units. Artificial neurons are basically processing units that compute some operations over several input variables and usually have one output calculated through the activation function. Typically, an artificial neuron has a weight ${w}_{i}$ that represents the degree of connection between artificial neurons, some input variables ${x}_{i}$, and a threshold vector $b$. Mathematically, the total input and output of artificial neurons can be described as follows:

1 $$u=\sum_{i}{w}_{i}{x}_{i}$$

2 $$z=f\left(u+b\right)=f\left(\sum_{i}{w}_{i}{x}_{i}+b\right)$$

where $u$, $z$, $x$, $w$, and $b$represent the total input, output, input variables, weights, and bias, respectively. $f(\bullet )$ denotes an activation function: a nonlinear function such as a sigmoid, hyperbolic, or rectified linear function. We employed a rectified linear function as the activation function, and this function is referred to as the Rectified Linear Unit (ReLU). ReLU can be defined as follows:

3 $$f\left(u\right)=\mathrm{max}\left\{0,u\right\}=\left\{\begin{array}{c}u \left(u>0\right)\\ 0 \left(u\le 0\right)\end{array}\right.$$

A CNN consists of a convolutional layer and a pooling layer. The convolutional layer plays a role in capturing the features from the images. In this process, a fixed-size filter runs over the images and extracts the patterns of shades of colors in the images. After each convolutional layer, there are pooling layers that are created to reduce the variance of features; this is accomplished by computing some operations of a particular feature over a region of the image.

The pooling layer has two functions. The first function is to reduce the position sensitivity of the feature that is extracted at the convolution layer so that the output amount of the pooling layer does not change even when the position of the feature amount extracted by the convolution layer is shifted within the image. The second function is to enlarge the receptive field for the following convolutional layers. Two operations may be realized on the pooling layers, namely, max and average operations, in which the maximum and mean values are selected over the feature region, respectively. This process ensures that the same results can be obtained even when image features have small translations or rotations, and this is crucial for object classification and detection. Thus, the pooling layer is responsible for sampling the output of the convolutional layer and preserving the spatial location of the image, as well as selecting the most useful features for the next layers. After several convolutional and pooling layers, there are fully connected layers, which take all neurons in the previous layer and connect them to every single neuron in its layer.

Finally, following all the convolution, pooling, and fully connected layers, a classifier layer may be used to calculate the class probability of each image. We employed the softmax function in this layer. The softmax function calculates the probabilities of each target class over all possible target classes and is written as follows:

4 $${y}_{k}={\mathrm{softmax}}_{k}\left({u}_{1},{u}_{2},\cdots ,{u}_{K}\right)=\frac{{e}^{{u}_{k}}}{\sum_{j=1}^{K}{e}^{{u}_{j}}}$$

where $k$ represents the number of output units and $u$ represents input variables.

To evaluate the performance of the network, a loss function needs to be defined. The loss function evaluates the effectiveness of the network in modeling the training dataset. The objective of training is to minimize the error of the loss function. The cross entropy of the softmax function is defined as follows:

5 $$E=-\sum_{n=1}^{N}\sum_{k=1}^{K}{t}_{n,k}\mathrm{log}{y}_{k}$$

where $t$ denotes the vector for the training data, $K$ represents the possible class, and $N$ represents the total number of instances.

### Approach

**Fig. 3:**
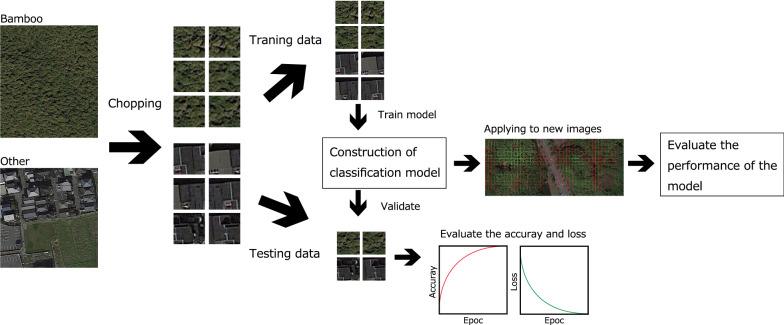
Outline of the approach adopted in this research. This figure was generated using data from Google Earth images (Image data: ^©^2018 CNES/Airbus & Digital Globe)

A schematic of our approach is shown in Fig. 3. We prepared the training data by using the chopped picture method [17]. First, in this method, we collected images that were (a) nearly 100% covered by bamboo and (b) not covered by bamboo. Next, we “chopped” these images into small squares with 50% overlap both vertically and horizontally. Finally, we used the chopped images as training images. Details about the size and number of training images for each study site are presented in Table 1.

**Table 1:** Size and number of training images used in each experiment

Zoom level	1/500			1/1000	1/2500
Chopping size (pix)	28	56	84	28	14
Sanyo-Onoda	67398	16207	6866	16000	16000
Ide	79043	18653	2836	18000	18000
Isumi	64106	15083	2837	15000	15000

We created a model for image classification from a CNN for bamboo forest detection. As opposed to the conventional approaches for training classifiers with hand-designed feature extraction, the CNN learns the feature hierarchy from pixels to classifiers and trains layers jointly. We used the final layer of the CNN model to detect the bamboo coverage from Google Earth images. First, we randomly shuffled all images to avoid overlapping of the training data and validation data. Then, we used 75% of the obtained images as training data and the remaining 25% as validation data.

We used the LeNet network [22], which is a classical deep learning model, because this study uses small-size images as the training and validation data. This network is constructed with two convolutional layers, two pooling layers, and a fully connected layer. The network architecture is discussed in Table 2. The model parameters implemented in this study included the number of training epochs (30), learning rate (0.01), train batch size (64), and validation batch size (32).

**Table 2:** Architecture of the network used in this study

Layer name	Filter size	Number of output neurons
Convolution 1	5 × 5	20
Pooling 1	2 × 2	
Convolution 2	5 × 5	50
Pooling 2	2 × 2	
Fully connected 1		500
Fully connected 2		2

### Evaluation of learning accuracy

The model was validated in each learning epoch by using the accuracy and loss functions. “Accuracy” indicates the accuracy of the model in classifying the validation images, whereas “loss” represents the inaccuracy of prediction by the model. If model learning is successful, loss (val) is low and accuracy is high. However, if loss (val) becomes high during learning, this indicates over fitting.

### Evaluation of model performance

To evaluate the performance of the model, we used the confusion matrix. First, we obtained 20 new images, which were uniformly covered by bamboo forest or objects other than bamboo forest, from each study site. In addition, 20 new images were sampled from multiple locations that were at least 5 km from the locations where the training images for each study site were sampled from. Second, we re-sized the images by using the chopped picture method without overlap. Third, we randomly sampled 500 images from the re-sized images. Fourth, we applied the model to the sampled images and evaluated the classification accuracy. Finally, we categorized the classification results into four groups: true positive (*TP*), false positive (*FP*), false negative (*FN*), and true negative (*TN*). Next, we calculated the classification accuracy, recall rate, and precision rate by using the following equations:

6 $$\text {Classification \; accuracy} = (TP+TN)/(TP+TN+FP+FN)$$

7 $$\text {Recall \; rate} =TP/(TP+FN)$$

8 $$\text {Precision\;rate}=TP/(TP+FP)$$

### Influence of image resolution on the classification accuracy

To quantify the influence of image resolution on the accuracy of detection, we constructed a model that correspond to image resolution. We obtained images at a zoom level of 1/500 (spatial resolution: ~ 0.13 m/pixel), 1/1000 (spatial resolution: ~ 0.26 m/pixel), and 1/2500 (spatial resolution: ~ 0.65 m/pixel) from each study site. Next, we applied the chopped picture method. To adjust the spatial extent of each chopped image, we chopped 56, 28, and 14 pixels for the 1/500, 1/1000, and 1/2500 levels, respectively. After constructing the model, we applied it to the 500 new images and calculated the classification accuracy, recall rate, and precision rate.

### Influence of chopping grid size on the classification accuracy

To quantify the influence of spatial extent of the chopping grid on the accuracy of detection, we chopped the 1/500 resolution images (spatial resolution: ~ 0.13 m/pixel) at each study site for three types of pixel sizes (84, 56, 28). After constructing the model, we applied it to new images and calculated the classification accuracy, recall rate, and precision rate. Note that our method does not perform dense prediction; therefore, the resolution of the final image will be lower than that of the original image. We considered this to be acceptable for the purpose of this study, because a previous study [23] has reported that more than 90% of the bamboo forests are distributed as patches of size greater than 5000 m^2^ in Japan.

### Transferability test

Given the large variations in the visual appearance of bamboo forest across different cities, it is of interest to study to what extent a model trained for one geographical location can be applied to a different geographical location. As such, we conducted experiments in which we trained a model for one (or more) city and applied it to a different set of cities. To test the transferability, we used 10 new 1/500 resolution images, and evaluated the performance of the model based on the classification accuracy, recall rate, and precision rate.

### Comparison with existing machine learning method

We compared the classification performance of our method with that of support vector machine (SVM), a supervised nonparametric classification algorithm. The SVM classification algorithm is commonly employed in remote sensing for a range of applications [24]. The SVM classifier tries to find the optimal hyperplane in *n*-dimensional classification space with the highest margin between classes.

In this experiment, we provided 56 pixel-chopped 1/500 resolution images for each study site as training images. Then, we applied the model to newly sampled 500 images and evaluated the classification accuracy, recall rate, and precision rate. We used RGB value and object-specific texture measures based on grey-level co-occurrence matrix (GLCM) as an image feature for SVM. To calculate the GLCM texture, we first calculated the luminance value of the image, which is accomplished by defining the weights for R, G, and B. Then, five GLCM texture measures of mean, variance, contrast, homogeneity, and dissimilarity were calculated. We employed RBF kernels for SVM classification and determined the parameters (gamma and cost) by a grid search method.

### Robustness assessment

We evaluated the classification performance of our method using different amounts of training data. In this experiment, we provided 15000, 1000, and 100 pixel-chopped images for each study site as training images. Then, we evaluated the classification accuracy, recall rate, and precision rate using 500 images that were randomly sampled from the images used in model performance evaluation.

## Results

### Fluctuation of accuracy and loss during the learning epochs

**Fig. 4:**
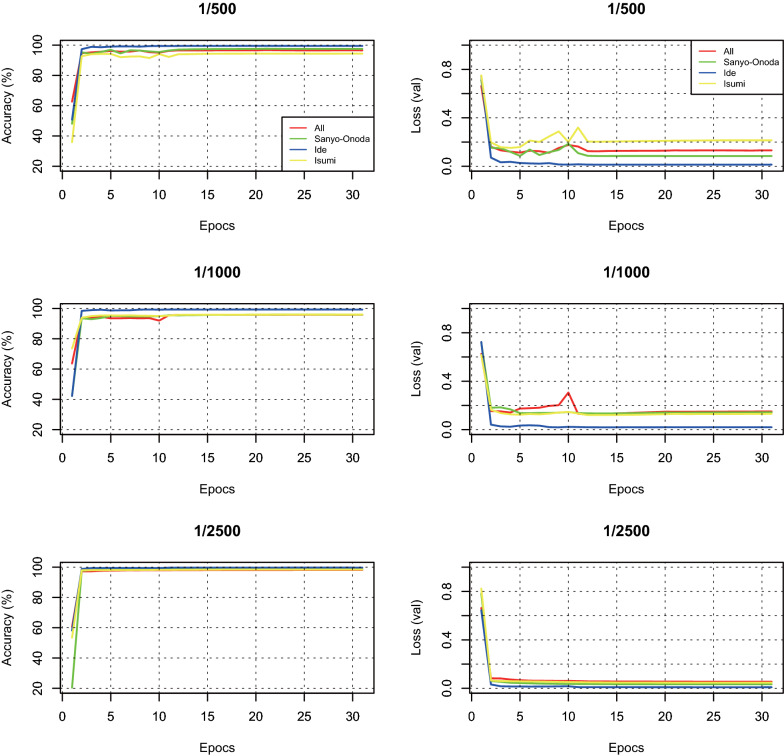
Accuracy and loss (val) of the model at each learning epoch

The accuracy for classifying the validation data of the final layer ranged from 94 to 99%, with an average of 97.52%. The loss values for the validation data ranged from 0.008 to 0.214, with an average of 0.086 (Fig. 4). The values of accuracy increased and those of loss decreased following the learning epochs (Fig. 4). These results suggest that all the models were not overfitted to the datasets and successfully learned the features of chopped pictures.

### Influence of image resolution on the classification accuracy

**Fig. 5:**
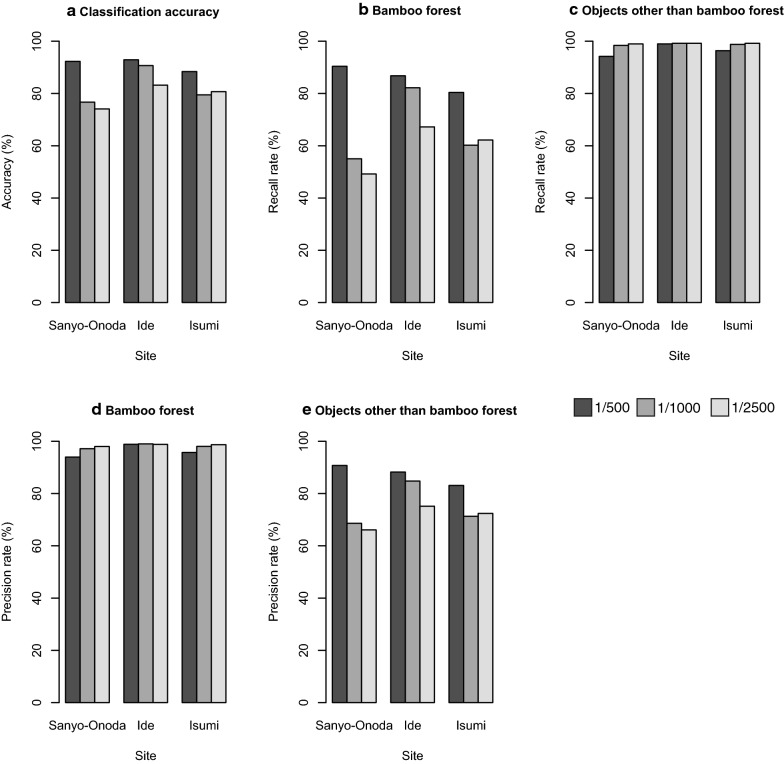
Sensitivity of the image scale versus the test accuracy

The classification accuracy ranged from 74 to 93% (Fig. 5a). The recall rate and precision rate for bamboo forest ranged 49% to 90% and 94% to 99%, respectively (Fig. 5b, d), and those for objects other than bamboo forest ranged from 94 to 99% and 66% to 91%, respectively (Fig. 5c, e). The classification accuracy and recall rate for bamboo forest declined following the decline in the image resolution (Fig. 5a, b).

### Influence of chopping grid size on the classification accuracy

**Fig. 6:**
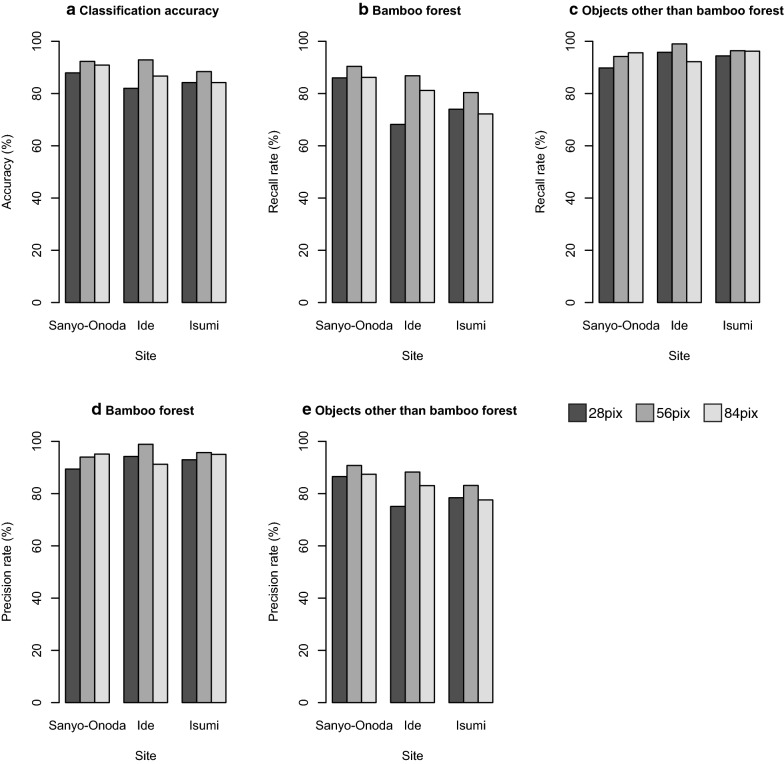
Sensitivity of the pixel size versus the test accuracy

The classification accuracy ranged from 82 to 93% (Fig. 6a). The recall rate and precision rate for bamboo forest ranged from 72 to 90% and 89% to 99%, respectively (Fig. 6b, d), and those for objects other than bamboo forest ranged from 89 to 99% and 75% to 91%, respectively (Fig. 6c, e). The intermediate-size images (56 pixels) exhibited the highest classification accuracy for all sites (Fig. 6a).

### Comparison with SVM

**Fig. 7:**
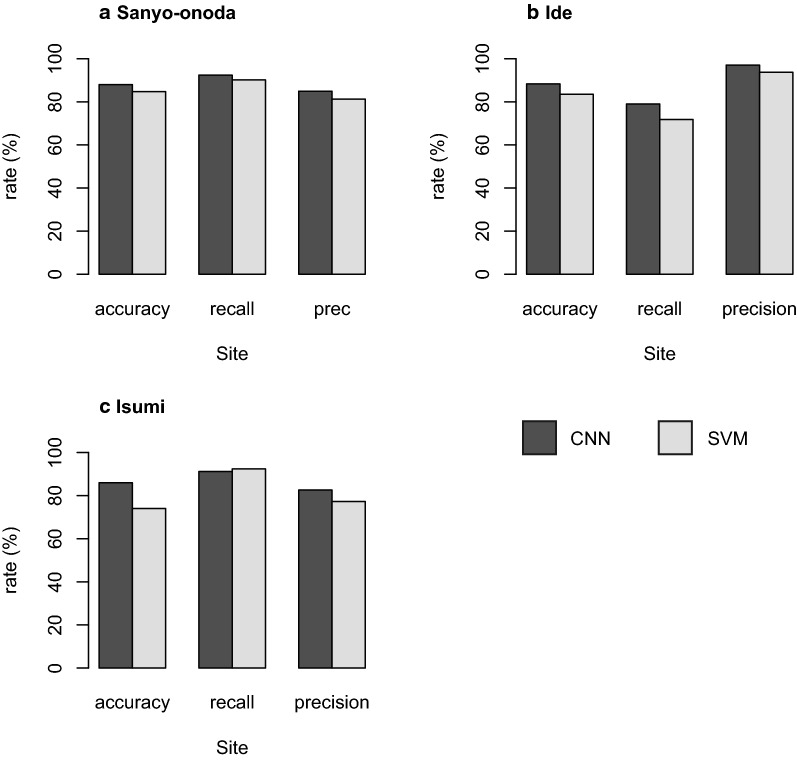
Comparison of classification performance between CNN and SVM

Our results show that the CNN classifier outperformed the SVM classifier (Fig. 7). The classification accuracy of CNN ranged from 86 to 88%, whereas that of SVM ranged from 74 to 85% (Fig. 7). The recall rate of CNN ranged from 79 to 92%, whereas that of SVM ranged from 71 to 91% (Fig. 7). The precision rate of CNN ranged from 82 to 97%, and that of SVM ranged from 77 to 93% (Fig. 7).

### Robustness assessment

**Fig. 8:**
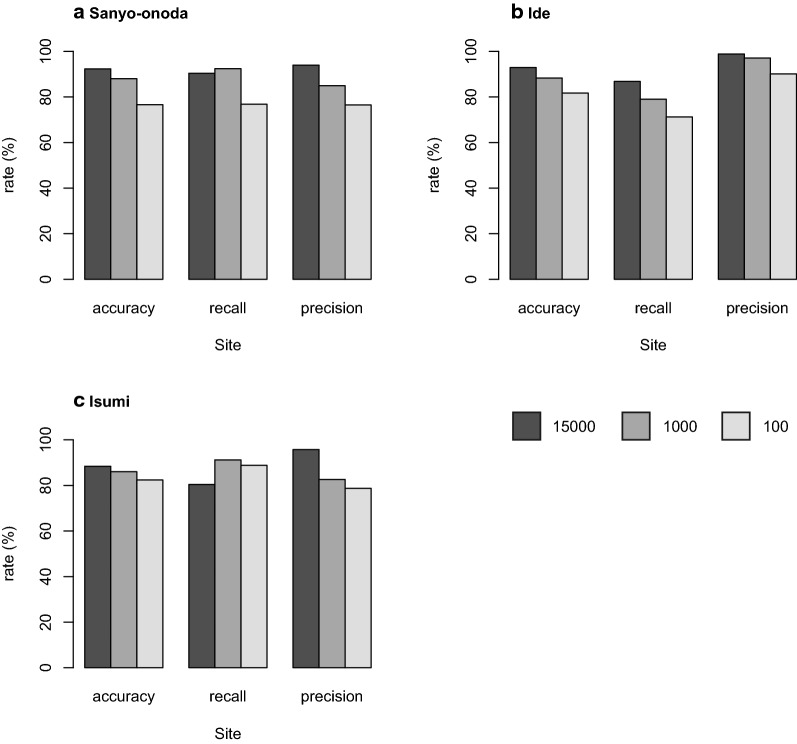
Evaluation of classification performance of our model with different amounts of training images

The classification accuracy of the model trained with 15000 images ranged from 88.4% to 92.9% (Fig. 8). The recall rate and precision rate for bamboo forest ranged from 80.4% to 90.4% and 93.9% to 98.8%, respectively (Fig. 8). The classification accuracy of the model trained with 1000 images ranged from 86 to 88% (Fig. 8). In this case, the recall rate and precision rate for bamboo forest ranged from 79% to 92.4% and 82.6% to 97.0%, respectively (Fig. 8). The classification accuracy of the model trained with 100 images ranged from 76.6% to 82.4% (Fig. 8), in which case the recall rate and precision rate for bamboo forest ranged from 71.2% to 88.8% and 76.4% to 90.1%, respectively (Fig. 8). Note that even when the number of training images was decreased to 100, a classification accuracy of greater than 75% was maintained.

### Transferability and classification performance

**Fig. 9:**
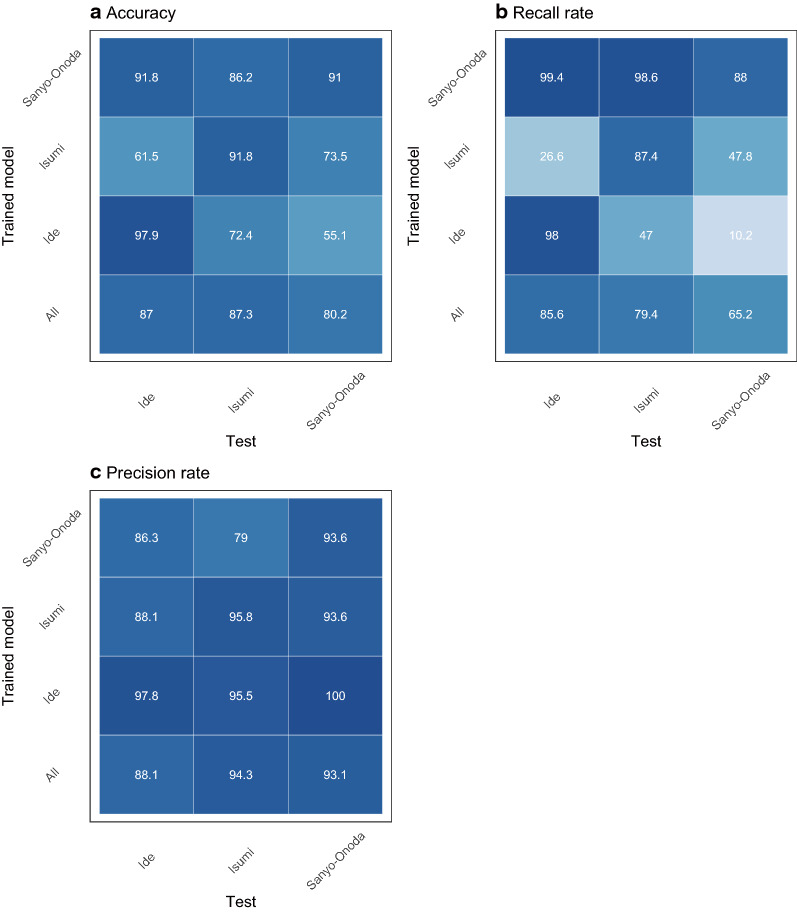
Transferability of the models trained for one location, but applied at another

In general, the performance of the model was poor when it was trained with samples from a given city and tested with samples from a different city (Fig. 9a). When the model trained with the images of Isumi city was applied to the other cities, the recall rate was the worst (Fig. 9b). Conversely, the model trained with the images of Sanyo city had the highest recall rate (Fig. 9b). We noticed that a more diverse set (all) did not yield a better performance when applied at different locations, compared with the models trained on individual cities (Fig. 9).

## Discussion

**Fig. 10:**
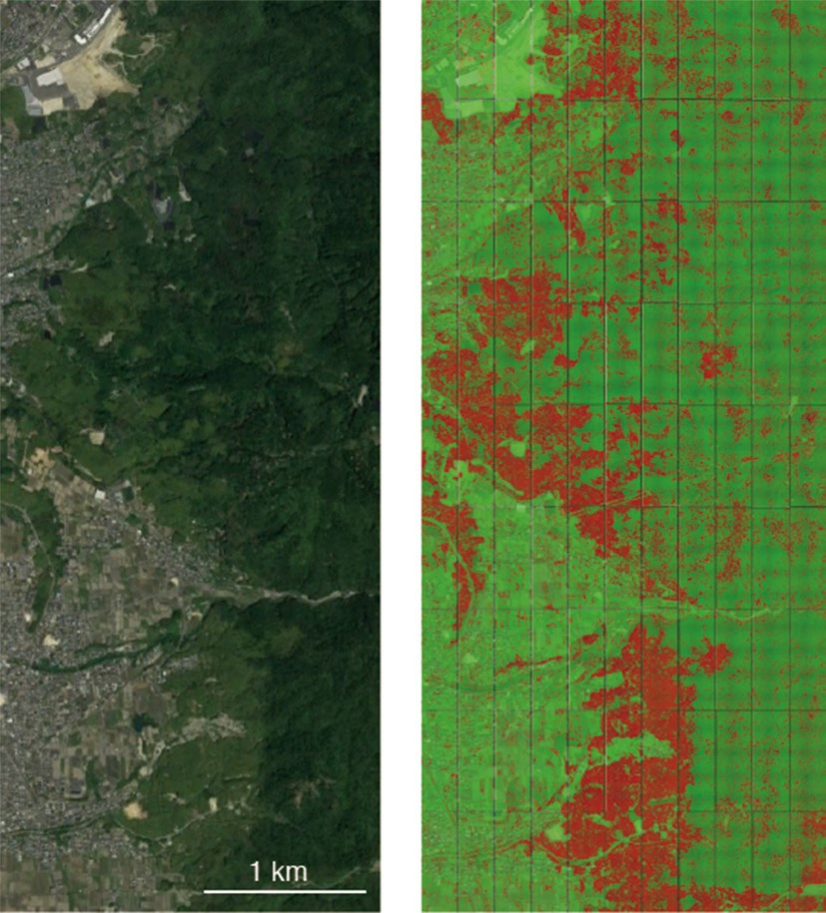
Example of applying the model to the wide area of Ide city. The left image is the original Google Earth image, and the right image shows the results of bamboo forest detection. Bamboo forests are highlighted by red, and objects other than bamboo are highlighted by green. This figure was generated using data from Google Earth image (Image data: ^©^2018 CNES/Airbus & Digital Globe)

In this paper, we demonstrated that the chopped picture method and CNN could accurately detect bamboo forest in Google Earth imagery. Recent studies [13, 14] have shown that the use of CNN for processing of color (RGB) images of the Earth surface yield high-accuracy results in the recognition of different tree species. However, most of the existing studies are aimed at identifying tree species at the individual tree level and cannot be applied to the detection of vegetation with ambiguous and amorphous shapes such as clonal plants. In this study, we employed the chopped picture method to detect bamboo forest from Google Earth images, and our results demonstrated good performance even though we employed the most classical CNN (LeNet). Additionally, our method outperformed SVM (Fig. 7), suggesting that the chopped picture method and CNN would be powerful methods for high-accuracy image-based bamboo forest detection and vegetation mapping (Fig. 10).

### Influence of image resolution on the classification accuracy

Our results indicate that the image resolution strongly affects the identification accuracy (Fig. 5). As the resolution rate decreased, the performance of the model also declined.

Specifically, in the 1/2500 imagery, the recall rate for bamboo forest of Sanyo-Onoda and Isumi city declined to 59% and 68%, respectively (Fig. 5b). In contrast, the precision rate for bamboo forest increased as the resolution decreased (Fig. 5d); this result indicates that as the resolution decreases, the model overlooks many bamboo forests. Thus, when the image resolution is low, it is difficult to learn the features of the object, and our approach is critically dependent on high-resolution data, with even the 0.65 m spatial resolution data being insufficient for producing the high-quality results. This result also suggests that in the deep learning model, misidentification due to false negatives is more likely to occur than misidentification due to false positives as the image resolution decreases.

### Influence of chopping grid size on classification accuracy

Our results indicate that the chopping grid size also affects the performance of the model. The classification accuracy was the highest at the medium pixel size (56 × 56 pixels; Fig. 6a). In contrast to the effects of image resolution, the recall rate and precision rate for bamboo forest was also the highest at medium pixel size, except for the recall rate for Ide city (Fig. 6b, d). This result indicates that if the grid size is inappropriate, both false positives and false negatives will increase.

Increasing the chopping grid size results in an increase in the number of chopped pictures, in which bamboo and objects other than bamboo are mixed. In this study, because we evaluated the performance of the model by using images that were uniformly covered by bamboo forest or objects other than bamboo forest, the influence of imagery consisting of mixed objects on the classification accuracy could not be evaluated. We will evaluate the classification accuracy for such images in our future research.

### Transferability among the models

The results of the transferability tests show that transferability was generally poor, and they suggest that the spatial extent of acquisition of the training data strongly influences the classification accuracy (Fig. 8). The model trained by Sanyo-Onoda city images yielded high recall rates for the images acquired at all study sites; however, the precision rate was lower than that of the other models (Fig. 8b, c). This means that the model trained by Sanyo-Onoda city images tends to produce false positive errors. Interestingly, transferability was not found to be related to the distance among the study sites (Fig. 8). This result indicates that the classification accuracy across the model reflects the conditions at the local scale, such as the weather conditions at the time when the image was acquired. Additionally, even when we applied a model that learned from all training images, the performance of the model was not as good as when the training data were obtained from the same city. The same tendencies have been reported in studies that classified land use by using deep learning [25]. This suggests that increasing the quantity of training data could cause a decrease in the identification accuracy, and it may be difficult to construct an identification model that is applicable to a broad area.

## Conclusion

We demonstrated that the deep learning model presented herein can detect bamboo forest from Google Earth images accurately. Our results suggested that the CNN technique and the chopped picture method would be powerful tools for high-accuracy image-based vegetation mapping, and exhibit great potential for reducing the efforts and costs required for vegetation mapping as well as improving the current status of monitoring the distribution of bamboo. Recently, bamboo expansion has become an important problem in Japan because of its invasiveness [18]. Although some studies have analyzed the bamboo forest distribution probability on a national scale [26, 27], monitoring of bamboo expansion is still challenging because it is labor-intensive. Nonetheless, our method has a certain degree of robustness in reducing the amount of training data (see Fig. 8). Therefore, we conclude that our approach could potentially lead to the creation of a semi or even fully automated system for monitoring bamboo expansion. Our results also suggest that the identification accuracy depends on the image resolution and chopping grid size; especially, the spatial resolution of training data strongly affects the model performance. Although satellite-based remote sensing has been widely studied and applied, it still has problems such as insufficient information due to low-resolution images or inaccurate information due to local weather conditions [28]. A possible way to overcome such difficulties is to use higher resolution satellites such as WorldView–4. It is expected that the classification accuracy of our approach will be further improved by using even deeper neural networks and images obtained by higher resolution satellites such as WorldView–4 in the future.

## Data Availability

This study was a re-analysis of existing data that are publicly available. Data on the Google Earth images openly available at https://earth.google.com/web/. The Caffe deep learning framework can be accessed at http://caffe.berkeleyvision.org/. The glcm packages and e1071 package [29] in R were used for calculating the GLCM texture and SVM implementation. These packages can be accessed at https://cran.r-project.org/web/packages/glcm/index.html and https://cran.r-project.org/web/packages/e1071/index.html

## Abbreviations

CNN: Convolutional neural network
SVM: Support vector machine
